# Short Interruptions of Imposed Hyperopic Defocus Earlier in Treatment are More Effective at Preventing Myopia Development

**DOI:** 10.1038/s41598-019-48009-3

**Published:** 2019-08-07

**Authors:** Alexandra Benavente-Perez, Ann Nour, David Troilo

**Affiliations:** SUNY College of Optometry, New York, NY USA

**Keywords:** Visual system, Experimental models of disease

## Abstract

The purpose of this study was to evaluate the effect of interrupting negative lens wear for short periods early or late during the development of lens-induced myopia in marmosets. Sixteen marmosets were reared with a −5D contact lens on their right eye (plano on contralateral eye) for 8 weeks. Eight marmosets had lenses removed for 30 mins twice/day during the first four weeks (early interruption) and eight during the last four weeks (late interruption). Data were compared to treated controls that wore lenses continuously (N = 12) and untreated controls (N = 10). Interocular differences (IOD) in vitreous chamber (VC) depth and central and peripheral mean spherical refractive error (MSE) were measured at baseline and after four (T_4_) and eight (T_8_) weeks of treatment. Visual experience during the interruptions was monitored by measuring refraction while marmosets were seated at the center of a 1 m radius viewing cylinder. At T_4_ the eyes that were interrupted early were not different from untreated controls (p = 0.10) and at T_8_ had grown less and were less myopic than those interrupted later (IOD change from baseline, VC: +0.07 ± 0.04 mm vs +0.20 ± 0.03 mm, p < 0.05; MSE: −1.59 ± 0.26D vs −2.63 ± 0.60D, p = 0.13). Eyes interrupted later were not different from treated controls (MSE, p = 0.99; VC, p = 0.60) and grew at the same rate as during the first four weeks of uninterrupted lens wear (T_4_ − T_0_: 3.67 ± 1.1 µm/day, T_8_ − T_4_: 3.56 ± 1.3 µm/day p = 0.96). Peripheral refraction was a predictive factor for the amount of myopia developed only when the interruption was not effective. In summary, interrupting hyperopic defocus with short periods of myopic defocus before compensation occurs prevents axial myopia from developing. After myopia develops, interruption is less effective.

## Introduction

Visual cues regulate emmetropization and can change the rate at which the eye grows^1–5^. This ability to regulate eye growth and refractive state does not require input from the brain^6,7^ and is locally controlled within the eye^8–11^. Studying the interactions between the spatial and temporal integration of visual signals across the retina and over time is crucial to understand how signals across the visual field, and the time spent on different visual tasks, affect emmetropization and refractive error development. This in turn will help develop more effective approaches to control myopia development and progression.

Visual experience influences refractive development in a variety of species. Depriving chicks, mammals and primates of form vision is known to elongate the eye and produce myopia^1,5,7,12–18^. Furthermore, restricting the visual deprivation to part of the visual field leads to increased eye growth and myopia development localized to the region of the eye that was deprived^8^. Image quality, not just visual deprivation, plays an important role in the control of eye growth. Modifying the spatial frequency and contrast properties of the visual stimuli using translucent diffusers in chicks, guinea pigs and rhesus monkeys leads to varying degrees of eye growth and myopia development, and requires loss of mid-spatial frequencies for significant effects^19–24^.

Optical defocus can also trigger changes in eye growth. It affects retinal image quality, can interfere with the contrast and spatial frequency properties of the retinal image, and affects emmetropization in a sign-dependent manner. In a variety of species, including non-human primates, imposing hyperopic or myopic defocus on the retina using negative or positive lenses respectively, results in compensatory eye growth that moves the retina to the imposed focal plane and corrects the imposed refractive error^3,16,25–29^.

The amount of compensation to lens-induced defocus of different sign is not symmetric. Tree shrews and primates compensate for imposed hyperopic defocus more completely than to equal amounts of imposed myopic defocus^27,30^. However, myopic defocus dominates hyperopic defocus when the stimuli are presented individually^31,32^, successively^33–35^ or simultaneously in various species including human^36–39^. A relatively small amount of alternated or simultaneous myopic defocus can cancel the compensation of greater amounts of hyperopic defocus^34,36,40,41^, and interrupting hyperopic defocus for even brief periods of time is also known to reduce myopia development in chicks, tree shrews and primates^26,35,42^.

The sign, frequency, and duration of imposed defocus are important factors that affect compensatory eye growth and refractive change, and may represent how real-world visual experience influences eye growth and the development of refractive state^43^. Studies of the temporal integration of defocus signals provide evidence for non-linear integration of the visual signals^31,40,44,45^. For example, in terms of the sign of defocus, the retina is more sensitive to myopic than to hyperopic defocus as shown by the preferred compensation to myopic defocus when both myopic and hyperopic defocus are presented simultaneously^36–39^. In terms of temporal frequency, multiple brief episodes that stimulate eye growth are more effective than longer less frequent episodes of the same total duration^44,46^ - a total of 28 mins/day of hyperopic defocus given 2 min/hr can induce increased eye growth in chick eyes whereas the same treatment duration given in 7 minute-periods every 3 hrs is not effective^44^. The time required to integrate the visual signal for compensatory eye growth depends on the sign of the defocus. In chicks, the axial and choroidal responses to either hyperopic or myopic defocus rises and saturates in less than 5 minutes^40^. However, the effect of myopic defocus takes longer to decline (24 hrs) than the effect of hyperopic defocus (20 mins), and the choroidal response to imposed defocus is also asymmetrical^40^. In guinea pigs, 30 mins of exposure to hyperopic defocus triggers 50% of the maximum myopic growth, and episodes of unrestricted vision as brief as 30 mins can interrupt the response^33^. Macaques and tree shrews show reduced myopia development when imposed hyperopic defocus from negative lenses is alternated with unrestricted vision compared with alternation with positive lens defocus or full time wear^34,35^.

How the eye integrates retinal defocus signals to control eye growth and refractive state is important for optimizing optical treatments of myopia^47^. In this study using a non-human primate (NHP) model of eye growth and myopia, we examined the effect of interrupting imposed hyperopic defocus with short daily periods of clear vision at either the beginning of treatment or later in treatment. We did this by evaluating the effect that the interruptions had on eye growth, central and peripheral refractive development, and other biometric changes, while measuring and controlling the visual experience and refractive state during the interruption periods.

## Materials and Methods

### Experimental protocol

Thirty-eight juvenile marmosets (*Callithrix jacchus*) were randomly assigned into two control and two treatment groups. Twelve marmosets wore −5D contact lenses continuously (uninterrupted treated controls). Ten marmosets were age-matched untreated controls (untreated controls). Two treatment groups wore −5D contact lens interrupted for 30 mins, twice a day. Eight marmosets had lens wear interrupted during the first four weeks of treatment and wore lenses continuously during the last four weeks of treatment (early interruption group). Eight marmosets wore lenses continuously during the first four weeks of treatment and had interruptions during the second four weeks of treatment (late interruption group). All animal care, treatment and experimental protocols were reviewed and approved by the SUNY College of Optometry Institutional Animal Care and Use Committee and conformed to the ARVO Statement for the Use of Animals in Ophthalmic and Vision Research.

All animals treated with contact lenses were reared with a full field negative (−5D) soft contact lens on their right eye (experimental eye), and a plano lens on the fellow eye (control eye). Lens wear began at an average age of 70 days (71.56 ± 6.14 days) for eight weeks. Lenses were inserted at lights on (1000 lux) and removed at lights off each day (9 hours light/15 hours dark)^27,36,48^. Contact lenses had diameters of 6.0 mm or 6.5 mm, base curves of 3.6 mm or 3.8 mm, were made of methafilcon A (55% water content, DK: 17) and were fit 0.10 mm flatter than the flattest keratometry measurement and assessed using an ophthalmoscope^27,49^. No corneal complications were observed in any of the animals treated in this or earlier studies with marmosets^36,48^.

The early interruption and late interruption treatment groups had lenses inserted around 9am (±1 hr) and lights were turned on. Lenses were worn for 3 hrs, removed for 30 mins around noon (±1 hr), worn for another 3 hrs, removed for 30 mins around 3 pm (±1 hr), and worn again for 3 hrs before removing lenses for the night and turning lights off around 6 pm (±1 hr).

**Figure 1:**
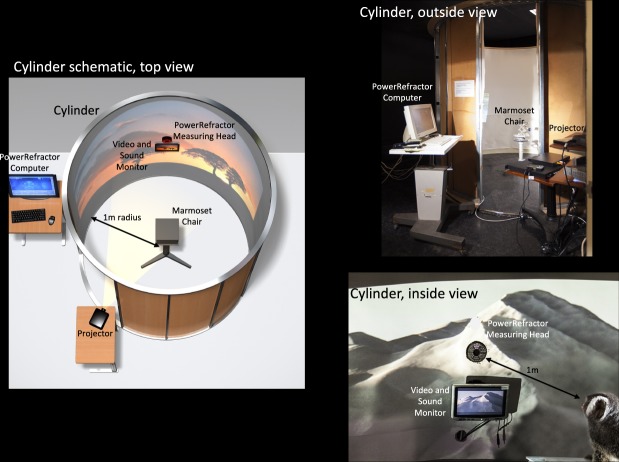
Schematic of the viewing cylinder used during the interruption periods. Gaze, fixation, and axial refractive state were measured with the IR video photorefractor. *(left)* Cylinder set up rendering, *(right)* actual photos of the set up. Photos and rendering copyright of William Bourassa Jr.

The typical 30 min interruption period began with contact lenses removal (1 min), transportation inside a dark nest box (5 min), seating in a primate chair located in the center of a viewing cylinder with a 1 m radius (5 min), transportation back to the animal facility (5 min) and lenses reinsertion (4 min). While the marmoset was inside the cylinder, they were gently restrained in a primate chair while were exposed to videos of natural scenes projected on the wall. During this time, the marmoset’s uncylopleged effective refractive state was measured every 5 minutes using an infrared video photorefractor (PowerRefractor, MultiChannel Systems, Tubingen, Germany). To capture the marmoset’s attention and successfully record their refraction, the video was turned off and a small monitor located below the photorefractor was turned on (Fig. 1, bottom right corner).

### Outcome measures

Ocular biometry and refractive state (mean spherical equivalent, MSE) were measured using our standard protocol for lens-treatment: we measured twice before treatment started (four weeks prior to lens rearing (T_−4_), again immediately before lens rearing (T_0_) and twice during treatment after four weeks (T_4_) and eight weeks (T_8_) of lens wear. Axial measurements of anterior chamber depth (ACD), lens thickness (LT), vitreous chamber depth (VC), choroidal thickness (CT), and retinal thickness (RT) were performed using high frequency A-scan ultrasound (25 MHz, Panametrics, NDT, Ltd, Waltham, MA). Central corneal curvature was measured by keratometry. On-axis refractive state was determined from the average of five measurements using the Nidek ARK-900 autorefractometer (Gamagori, Japan). All measures were performed 20 minutes after the instillation of 2 drops of 1% cyclopentolate and were completed within two hours.

Raw data are provided in supplemental tables. Data analysis and graphical representation are presented as interocular differences between the right treated eyes and the left contralateral eyes, normalized to baseline measures. Growth and refractive rates were calculated as the daily change in vitreous chamber and refraction during the first 4 weeks of treatment (early rate) and the second 4 weeks of treatment (late rate).

Peripheral refraction was measured continuously along the horizontal meridian using IR video photorefraction^36,48^. Peripheral refractive data was collected from 40° on the temporal retina to 40° on the nasal retina, using a running average of refraction data of approximately 500 refractions across the horizontal meridian. Relative peripheral refractions at 20° and 40° were calculated by subtracting the refraction on the nasal and temporal peripheral retina from the central refraction as previously described^36,48,50,51^.

Visual experience on the peripheral retina during the interruption periods was estimated by adding measures of peripheral refractive state taken during the T_4_ and T_8_ measures to the axial defocus experienced during the interruption on the first day of each four week interruption period. This provided an estimate of the effective peripheral refraction for the first day of each four week interruption period. The peripheral refractions were measured outside the viewing cylinder while the axial refractions were measured inside the cylinder and were adjusted for the 1D demand of the cylinder radius.

### Statistics

Stata (College Station, TX) was used to perform statistical analysis. One-way ANOVA and *post-hoc* analysis using Tukey tests were used to examine the differences between treatment and control groups. Repeated measures ANOVA was used to examine treatment effects over time within each group. Pearson’s linear correlation was used to explore the relationship between the effective peripheral refraction and the compensatory changes in refraction and eye growth. Multi-regression models were developed to evaluate whether central and peripheral refraction at baseline would predict the induced changes in growth rate after 4 (T_4_) and 8 (T_8_) weeks of treatment.

## Results

### Baseline measurements

There were no interocular differences in refractive state or any ocular dimensions measured in any of the groups prior to the beginning of treatment (supplemental material, Table S1).

### Overall treatment effects

Table S1 (supplemental material), summarizes the average ocular biometry and refractive state for the experimental and control eyes in each group at baseline, and after 4 and 8 weeks of treatment. Table S2 (supplemental material) describes these values as changes from baseline to four weeks, and from four to eight weeks.

**Figure 2:**
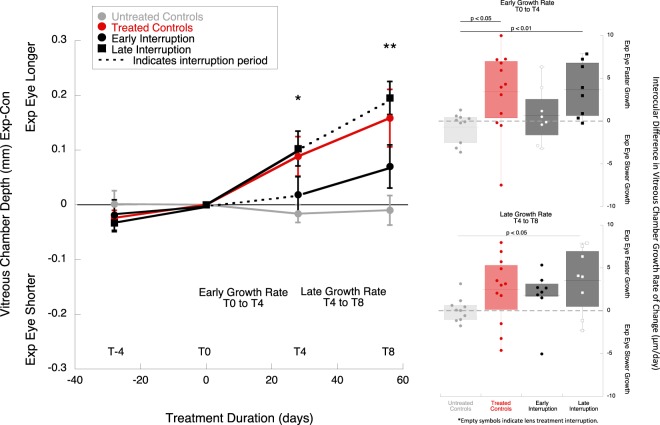
(*left*) Average interocular differences in vitreous chamber depth for the experimental and control eyes in each group, normalized to baseline (T_−4_: 4 weeks prior to treatment, T_0_: baseline, T_4_: 4 weeks of treatment, T_8_: 8 weeks of treatment). The early interruption group is represented by black circles, and the late interruption group by black squares. The interruption period is indicated with dotted lines. The results from these two treatment groups were compared to treated controls (continuous lens wear, red symbols) and untreated controls (grey symbols). * Indicates statistically significant ANOVA at p < 0.05, ** indicates statistically significant ANOVA at p < 0.01. *(right)* Box plots represent interocular changes in ocular vitreous chamber growth rate (exp-con) during the early (T_0_ to T_4_), and late treatment period (T_4_ to T_8_) for the different groups: treated controls (red), early interruption (black), late interruption (black) and untreated controls (grey). The data points from the interruption periods are indicated by empty symbols. The data are shown as mean ± SE.

**Figure 3:**
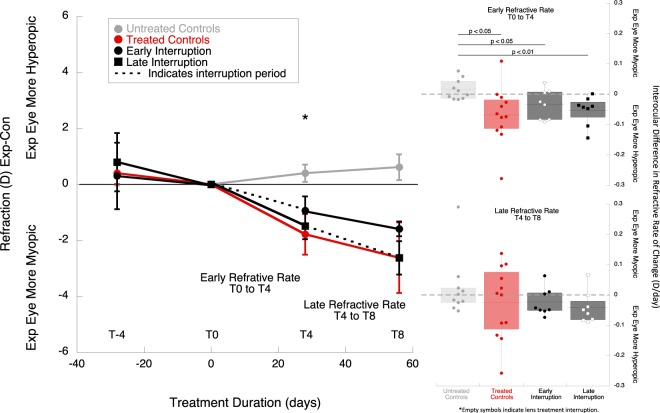
(*left*) Average interocular differences in refractive error for the experimental and control eyes in each experimental group, normalized to baseline. The early interruption group is represented by black circles, and the late interruption group by squares. The interruption period is indicated with dotted lines. The results from these two treatment groups were compared to treated controls (continuous lens wear, red symbols) and untreated contols (grey symbols). *(right)* Box plots representing the changes in interocular differences in refractive rate (exp-cont) during the early (T_0_ to T_4_), and late treatment period (T_4_ to T_8_) for the different treatment groups: treated controls (red), early interruption (black), late interruption (grey) and untreated control (no color). The interruption periods are indicated by empty symbols. The data shown are mean ± SE.

The treatment effects were calculated as the interocular difference (experimental eye minus control eye, exp-con) in vitreous chamber (VC, Fig. 2) and refractive error (mean spherical equivalent, MSE, Fig. 3), indicated as changes from baseline. At the end of the 8 weeks of treatment (T_8_), the treated eyes of the late interruption group and the treated controls were relatively larger and more myopic than untreated controls and the early interruption group (Fig. 2 *(left)* VC mean ± SE; early interruption group: +0.07 ± 0.04 mm; late interruption group: +0.20 ± 0.03 mm; treated control: +0.16 ± 0.05; untreated control: −0.01 ± 0.03; p < 0.01; ANOVA F = 4.81, Tukey post-hoc late interruption vs untreated p = 0.01, continuous lens wear vs untreated p < 0.05. Figure 3 *(left):* MSE early interruption: −1.59 ± 0.26D; late interruption: −2.63 ± 0.60D; treated control: −2.62 ± 1.26D; untreated control: +0.62 ± 0.46D; p < 0.05, ANOVA F = 3.13, Tukey post-hoc late interruption vs untreated p < 0.05, treated control vs untreated control p < 0.05). The refraction and vitreous growth in early interruption group did not differ statistically from untreated controls (p = 0.34 and p = 0.57, respectively).

No differences between groups were observed in corneal curvature or lens, retina and choroid thickness changes (ANOVA p > 0.05, Tables S1 and S2).

### Axial growth rates

During the first four weeks of treatment (T_0_ to T_4_), the interocular difference in vitreous growth rate was similar between the early interruption and untreated control groups (Fig. 2, top right panel, early interruption T_0_ to T_4_: 0.67 ± 1.1 μm/day, untreated: −0.68 ± 0.6 μm/day, ANOVA F = 3.60, Tukey post-hoc p = 0.84). Late interruption animals had faster ocular growth rates (+3.67 ± 1.1 μm/day, Tukey post-hoc p < 0.01). During the later four weeks of treatment (T_4_ to T_8_), the eyes of animals in the early and late interruption groups grew at similar rates (Fig. 2, bottom right panel early interruption group T_4_ to T_8_: 1.84 ± 1.07 μm/day; late interruption group: 3.56 ± 1.3 μm/day; ANOVA F = 1.88, Tukey post-hoc p = 0.71). During the later 4 weeks of treatment, the eyes of the late interruption group grew at a faster rate than untreated control animals (untreated 0.10 ± 0.44 μm/day, p < 0.05). The eyes that were interrupted during the last four weeks of treatment continued to grow at the same fast rate as they did during their first four weeks of uninterrupted lens wear (growth rate during first four weeks +3.67 ± 1.1 μm/day, last four weeks +3.56 ± 1.3 μm/day, p = 0.96).

### Refractive state rates

Interrupting imposed defocus early resulted in less myopia development compared to interrupting it later, or not interrupting at all, but these differences did not reach statistical significance (Fig. 3, top right panel, refractive change T_0_ to T_4_, early interruption: −0.03 ± 0.02D/day, late interruption: −0.05 ± 0.02D/day, treated controls: −0.11 ± 0.06D/day; ANOVA F = 0.58, p = 0.56). During the first weeks of treatment, the rates of refractive change of the early interruption, late interruption, and treated controls were all faster than the untreated controls (untreated: +0.01 ± 0.01D/day, all p < 0.05). During the second four weeks of treatment, the rate at which animals became myopic was slower in the early interruption group than the late interruption group, but this did not reach statistical significance (Fig. 3, bottom right panel refractive change T_4_ to T_8_, early interruption: −0.02 ± 0.02D/day, late interruption: −0.04 ± 0.0.02D/day, treated control: −0.02 ± 0.03D/day; untreated control: +0.02 ± 0.03D/day; ANOVA F = 0.90, p = 0.45).

### Visual experience during contact lens interruption

**Figure 4:**
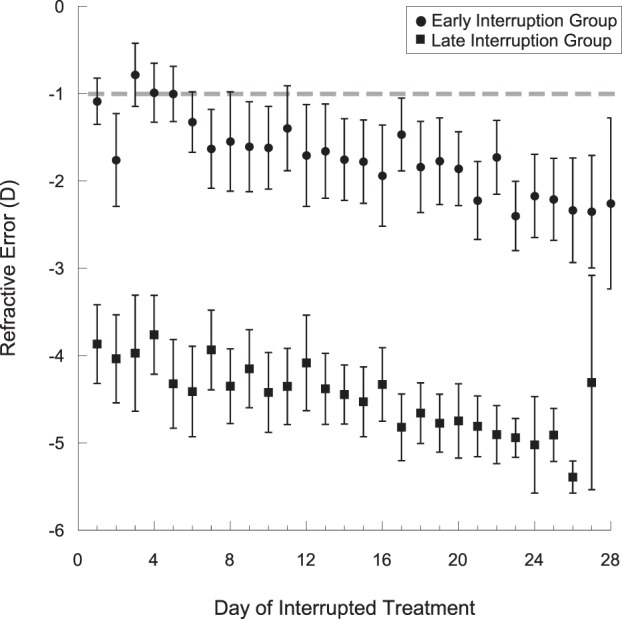
Daily average effective defocus experienced by the marmosets during the interruption periods while they were inside the viewing cylinder. The early interruption group is represented in black, and the late interruption group is represented in grey. The dotted grey line indicates the refraction at which the image on the cylinder wall would be focused on the marmoset retina, calculated from the cylinder dimensions. The data are shown as mean ± SE.

Marmosets in the early interruption group were treated for a total of 28 days from baseline to 4 wks. In the late interruption group, interruptions were over 27 days in two animals and 25 days in six animals. The defocus that marmosets experienced inside the viewing cylinder during the interruptions was, on average, myopia (Fig. 4, early interruption group: −1.72 ± 0.08D, late interruption group: −4.47 ± 0.08D, p < 0.05). There was a change towards greater myopic defocus by the end of treatment in both groups (early interruption: −1.08 ± 0.26D to −2.26 ± 0.98D, p < 0.05; late interruption: −3.87 ± 0.45D to −5.38 ± 0.65, p < 0.05).

**Figure 5:**
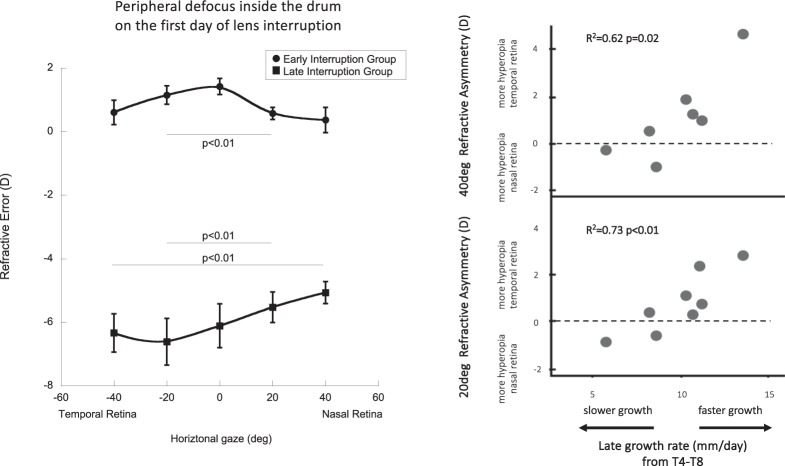
(*left*) Average peripheral refraction experienced by each treatment interruption group during the first day of treatment interruption; early interruption group shown by black circles; late interruption group shown by black squares. The data are shown as mean ± SE. *(right)* Multiple regression plotted as a matrix of two individual bivariate plots (NTasym40° R^2^ = 0.62 p = 0.02; NTasym20° R^2^ = 0.73 p < 0.01).

Marmosets in the early interruption group experienced axial hyperopic defocus during the first day of treatment interruption (Fig. 5, +1.42 ± 0.25D,) and relative myopia off-axis that was greater on the nasal than on the temporal retina (Fig. 5, +0.37D at 40 deg nasal, +0.61D at 40 deg temporal, p < 0.01). Marmosets in the late interruption group experienced central myopia during their first day of interruption (−6.10D), relative hyperopia on the nasal retina (−1.04D at 40 deg nasal) and relative myopia on the temporal retina (+0.33D at 40 deg temporal, p < 0.01).

### Predictors of lens compensation

When all treated animals were evaluated (early interruption, late interruption, and treated control groups) there was a trend in the relationship between the degree of the lens compensation achieved during the first four weeks and the number of hours that the animals had been wearing the contact lenses. Eyes got larger when the −5D contact lenses had been worn for 9 hrs without interruption during the first four weeks of treatment (linear regression between growth rates and treatment time, R = 0.44 p = 0.08).

We determined the effect that interrupting treatment had on the biometric changes of the anterior chamber depth (ACD), lens thickness (LT), choroidal thickness (CT), and retinal thickness (RT) (Tables S1 and S2). We used multiple regression analysis to assess how the eye changed as it grew faster or slower during the treatment conditions. During the first 4 weeks, treated control eyes grew more and had relatively larger ACDs and thinner choroids (multiple regression, R^2^ = 0.77 p < 0.05) compared to contralateral plano treated control eyes. Eyes that grew less with interrupted lens wear exhibited a relative thickening of the lens (multiple regression, R^2^ = 0.56 p < 0.05). During the second 4 weeks, the vitreous chamber of both treatment groups grew by the same amount (mean VC growth ± SE exp-con: 1.84 ± 1.07 µm/day vs 3.56 ± 1.35 µm/day, p = 0.33) and developed thinner retinas, thinner choroids, and deeper ACD (multiple regression, R^2^ = 0.84 p < 0.05).

**Figure 6:**
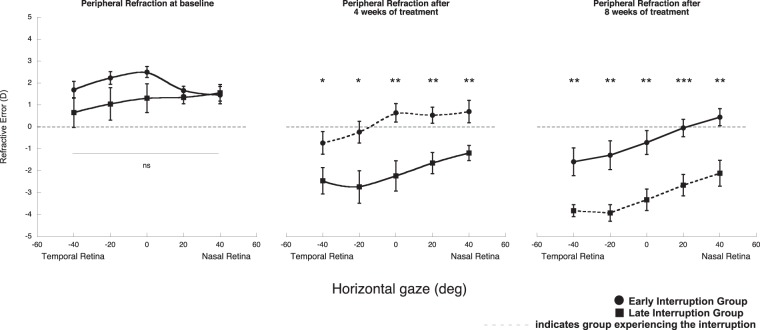
Average peripheral refraction experienced by the early and late interruption groups at baseline, after four weeks of treatment and after eight weeks of treatment. Early interruption group shown by black circles; late interruption group shown by black squares (ns, not clinically significant; *p < 0.05, **p < 0.01, ***p < 0.001). The data are shown as mean ± SE.

Both the early and late interruption groups experienced changes in peripheral refraction throughout treatment (Fig. 6). At baseline, the average peripheral refractive state did not differ statistically between groups, but after the first four weeks of treatment the early interruption group remained hyperopic on axis and on the nasal retina, while the temporal retina became myopic. At this time, the late interruption group had developed myopia on axis, and was relatively myopic on the nasal retina and relatively hyperopic on the temporal retina. Peripheral refraction at all retinal locations differed significantly between groups (Fig. 6; all p < 0.05). At the end of eight weeks of treatment, both the early and late interruption groups developed more myopia on axis, and the nasal-temporal (NT) asymmetry in their peripheral refractions became more pronounced. While the NT asymmetry did not differ between the groups, their peripheral refractions were significantly different at all retinal locations (Fig. 6; all p < 0.01).

Multiple regression analysis was used to assess whether the compensatory growth rates at each treatment period could be explained by pretreatment factors. Baseline peripheral refraction and early growth rates in all the groups combined did not correlate with the induced ocular growth changes at T4. However, we found that in the early interruption group, the naso-temporal asymmetries in relative peripheral refraction at 20° and 40° (NTasym20°, NTasym40°) at the end of the first four weeks of treatment was correlated with the growth rate during the later four weeks of treatment, such that faster growth rate was related to more positive NT asymmetries (that is, more relative hyperopia on the nasal side)(R^2^ = 0.81 p < 0.05). Figure 5 *(right)* describes the multiple regressions plotted as two individual bivariate plots (NTasym40° R^2^ = 0.62 p = 0.02; NTasym20° R^2^ = 0.73 p < 0.01). When both variables are combined in the multiple regression analysis, the regression value of the growth rate increases to R^2^ = 0.81 p < 0.05.

## Discussion

Short daily interruptions to imposed hyperopic defocus can affect the development of axial myopia significantly and slow down the compensatory vitreous chamber growth and refractive change. The effectiveness of the interruption  is associated with the treatment period; the earlier the interruption to imposed defocus occurs, the more effective it is at reducing the compensation for the lens-imposed defocus.

In this study, we interrupted imposed hyperopic defocus during the first or second four weeks of an eight-week lens-wear treatment. When imposed defocus was interrupted during the first four weeks of treatment, the axial growth and refractive rates were comparable to those of untreated control eyes and slower than when we interrupted later during treatment. When defocus was interrupted during the second four weeks of treatment, axial growth and refractive state were not different than uninterrupted controls with continuous lens wear. It was, therefore, more effective to interrupt imposed defocus during the first weeks of treatment before compensation had proceeded, than after the eye had compensated for approximately 70% of the imposed −5D defocus (−3.53D). Other than vitreous chamber depth, none of the other ocular parameters measured changed significantly throughout treatment. Our results support the idea that treating myopia before onset or as early as possible in development will be more effective in reducing progression. Guidelines regarding myopia control treatment in children before myopia onset, based on their risk factors, are being developed^52^.

Why interrupting imposed defocus later in treatment is ineffective may be related to greater myopia in those eyes, but the reasons are not clear. Greater myopic blur was experienced inside the viewing cylinder during the interruptions, but this is unlikely to be an important factor because comparable degrees of myopic defocus (+5D) in marmosets have been reported to induce hyperopia^27,36,48^. It also seems unlikely that the slightly older age of the marmosets would explain the ineffectiveness of the late interruption. In chicks, tree shrews, and non-human primates, form-deprivation myopia (FDM) has been induced in adolescent or adult animals with longer periods of form deprivation needed in the older animals, which suggests that the emmetropization mechanism remains active in adulthood but requires longer exposure periods^53–56^. In tree shrews^57^, the compensation to −5D lenses takes 10 days in younger animals, and 40 days in older animals. This suggests that the emmetropization mechanism for both FDM and lens-induced myopia (LIM) remains active into young adulthood, with younger animals developing larger amounts of myopia faster. We speculate that, because of the older age of the animals and the slowing of eye growth, a longer period of late interruption may be needed for a protective effect. In the tree shrew, similar to what was observed in this study, refraction state remained stable after compensating for −5D lenses. This is important because in this study marmosets treated for 8 weeks compensated for 75% of the imposed defocus during the first four weeks, after which the growth rate slowed down because they experienced less amount of defocus.

Imposed changes in refractive state across the retina are known to affect eye growth and refractive state development^11,36,37,48,58,59^ and may affect the effectiveness of the interruption to lens-imposed defocus as well. In this study, we found that in the uninterrupted controls, naso-temporal (NT) asymmetries in relative peripheral refraction at 20° and 40° after four weeks of treatment predicted growth rate during the second four weeks of treatment. Faster growth rate correlated with more positive NT asymmetries (more relative peripheral hyperopia on the nasal compared to the temporal retina). Peripheral refraction asymmetries have been associated with larger responses to imposed hyperopic defocus in marmosets^48^, and may partially explain the ineffectiveness of interrupting lens-induced myopia at the end of treatment.

It is also likely that the myopic visual signal interrupting the imposed hyperopic defocus was not strong enough to slow eye growth once myopia had developed. In this study we determined the defocus experienced by the retina during the treatment interruption. Work from other labs has described the effectiveness of interrupting lens-wear or form-deprivation myopia with short intervals of unrestricted vision^31,33,35,60^, but the actual refractive state during the interruption was not measured. In our study, the average effective defocus experienced during the contact lens interruption during the first weeks of treatment was mildly myopic. As animals compensated for imposed hyperopic defocus and developed more myopia throughout treatment, the effective defocus experienced during the interruption episodes also became more myopic, but the interruptions were not long enough or the signal was not strong enough to stop the myopia and reverse it. Marmosets treated with −5D lenses for 12 weeks can recover from the induced myopia and return to control levels within 3 months of removing lenses (unpublished data). The lens-interruption protocol presented here and the recovery protocol differ significantly and the results cannot be compared directly. However, the recovery observations suggest that more time or a stronger myopic visual signal is needed to reduce myopia once it has developed.

The early interruptions before myopia developed produced a small myopic visual signal that was sufficient to reduce compensation for the imposed hyperopic defocus. The average baseline refractive state of the marmosets in this study was slightly hyperopic, which is typical for marmosets at this age (10 weeks old)^27,36,48^. Treated eyes interrupted during the first four weeks of treatment were on average +1.31D, and their effective refraction without cycloplegia inside the viewing cylinder during the interruption was −1.66D, which was only slightly myopic (−0.66D) because the cylinder had a radius of 1 m (1D demand).

The effectiveness of replacing a myopia-inducing stimulus with varying amounts of myopic defocus has been studied in chicks, tree shrews, guinea pigs and macaques in the past with varying results (Table 1)^33,34,41,44,61^. In macaques, interrupting 12 hrs of hyperopic defocus (−3D) with plano lenses for 15 mins four times/day resulted in a 64% reduction in myopia development, whereas substituting the hyperopic defocus with myopic defocus (+4.5D) reduced myopia by only 46%. In tree shrews, removing negative lenses (−5D and −3D) for 45 mins/day blocked the myopic compensation, whereas substituting with positive lenses (+3D to +10D) increased myopia^34^. Interestingly, also in tree-shrews, replacing high-powered negative lenses (−9.5D) with a positive lens (+4D) was more effective than unrestricted vision; however, the animals could move freely during the interruption period so the actual refractive state was unknown^61^. In chicks, interrupting 9 hrs of negative lenses (−10D) with 3 hrs of normal vision^41^, or alternating 25 mins of −6D with 5 mins of +6D twice a day^44^, both blocked myopia development. Overall, these results suggest that the defocus detection threshold varies across species. Chicks appear more sensitive to myopic defocus than mammals, including non-human primates.

**Table 1:** Summary table of relevant studies across different species where negative lens wear was interrupted with periods of darkness, periods of free viewing or periods of positive lens wear.

Reference	Species	Degree of Imposed Defocus	Type of Interruption	Interruption Duration	Visual experience during the interruption	Study outcome
*Schmid and Wildsoet 1996*	Chicken	Monocular −10D, +10D or plano lens	Lens removed	1 × 11, 9, 6, 3 or 0 hrs/day	Unrestricted vision	Interrupting lens wear with periods of normal vision is more effective at reducing lens-induced myopia than at reducing lens-induced hyperopia.
*Winawer and Wallman 2002*	Chicken	Monocular −6D or +6D lens	Kept in the dark wearing lenses	Five dark periods: 1. 7 × 30 mins/day every 2 hrs 2. 58 mins/hour every hour 3. 13.5 hrs/day 4. 13.75 hrs/day 5. 4 × 30 mins/day	Kept in the dark with lenses on	Three main findings: 1. Several brief daily interrupting episodes are more effective than a single or a few longer daily ones. 2. Extremely brief episodes are ineffective. 3. When positive and negative lenses are worn successively, the positive lens has the dominant effect, even if the negative lens is worn five times longer.
*Norton et al*. *2006*	Tree Shrew	Monocular −5D lens	Lens removed and substituted for −5D, −3D, plano, +3, +4, +5, +6, or +10D	1 × 45 mins/day	Controlled distance in lab room with refractive monitoring	High degrees of myopic defocus (>5D) cannot compete with lens-induced myopia. Low degrees myopic defocus effectively reduced lens-induced myopia only in some eyes.
*Kee et al*. *2007*	Rhesus monkey	Binocularly −3D lens	Lens removed and substituted for plano or +4.5D	4 × 15 mins/day	Not mentioned in the paper	Brief periods of unrestricted vision prevented lens-induced myopia. Brief periods of viewing through +4.5D lenses produced weaker protective effects.
*McBrien et al*. *2012*	Tree Shrew	Binocular −9.50D	Lens removed and substituted for plano or +4D	1 × 1 hr/day or 2 × 30 mins/day	Unrestricted vision in an open-mesh cage	Interrupting negative lens wear with 1 hr of positive lens wear was more effective in preventing the development of experimentally induced myopia than interrupting using a plano lens
*Leotta et al*. *2013*	Guinea Pigs	Monocular −4D or −5D lens	Kept in the dark wearing lenses or lens removed	Dark period 1 × 0.25, 1, 2, 4 or 6 hrs/day Lens removed 1 × 5 mins, 15 mins, 30 mins, 1 hr, 3 hr, 6 hr, 9 hr/day	Kept in the dark with lenses on or Unrestricted vision with lenses removed in an open lab environment	Lens-induced myopia develops after brief exposures to hyperopic defocus and is very long lasting without competing signals. The induced myopia rapidly decays if interrupted by one 30 min period/day of ‘normal viewing’.

Several studies have identified non-linearities in the effect that imposed defocus has on the visual control of eye growth and refractive state^31,62,63^. Generally, imposed hyperopic defocus produces more compensatory myopia than imposed myopic defocus produces compensatory hyperopia^31,32^. Interrupting imposed hyperopic defocus with unrestricted vision is more effective at reducing compensatory myopia than using myopic defocus^34,64^. However, experimental eyes are more sensitive to myopic defocus and develop hyperopia when exposed to alternating myopic and hyperopic defocus, even if hyperopic defocus is experienced for longer^40^, or when they are presented simultaneously^36^. These apparent non-linearities are probably related to several factors including the degree of existing refractive error and differences in the ability to reduce or increase axial growth as it relates to existing growth rate. Spherical aberration and defocus interact to affect retinal image quality and the visual signal for emmetropization^65^, and might also explain differences in the efficacy of imposed defocus, as well as differences in the effects of unrestricted vision in eyes with varying amounts of myopia, to alter eye growth and refractive state.

Different results in published studies of temporal integration may relate to differences in the species and experimental protocols used. For instance, chick, tree shrews, guinea pig and macaque studies always interrupted the imposed hyperopic defocus from the beginning of treatment^33,34,41,44,61,64^. In this study lens rearing was interrupted either from the beginning of treatment or after the eyes had already compensated for 75% of the imposed defocus 4 weeks into treatment. Interrupting lens-wear after compensation had occurred in other species has not been reported. Studies performed in other species interrupted negative lens-rearing with periods of darkness, periods of free viewing or by replacing hyperopic for myopic defocus. In our study we interrupted lens-rearing with episodes of fixed visual space and monitored refraction during the interruption. Only one other study monitored refraction during the interruption^34^, they reported similar results to what was described here; interrupting exposure to hyperopic defocus with minimal defocus effectively reduces the development of compensatory eye growth and myopia^34^. The effectiveness of varying degrees of myopic defocus during interrupted hyperopic defocus has also been described in macaques, chicks and guinea pigs^35^.

## Conclusion

In this study we report that two 30-min interruption periods versus 8 hrs of imposed hyperopic defocus effectively blocked the compensatory eye growth leading to axial elongation and myopia, but only if the eye had not started to develop myopia. Because this study used only one power to induce myopia (−5D) and one interruption paradigm (2 × 30 mins episodes), we cannot determine at what degree of induced myopia the interruption loses effectiveness, nor can we determine whether there is a more effective interruption and visual stimulus to use once myopia has begun. Additional experiments examining the effects of different visual conditions during interruptions of lens imposed myopia are needed.

In summary, we found that brief periods of unrestricted vision can reduce the development of experimental myopia induced by lens-imposed hyperopic defocus. The effect was strongest when the interruption started at the same time as the lens rearing and when the eyes had only partially compensated for the imposed hyperopic defocus. These results suggest that optical treatments to reduce myopia progression may be more effective the earlier they are started.

## Supplementary information

Supplemental material Table 1

Supplemental material Table 2

## Data Availability

The datasets generated during and/or analyzed during the current study are available from the corresponding author on reasonable request.

## References

[1] Wiesel TN, Raviola E (1977). Myopia and eye enlargement after neonatal lid fusion in monkeys. Nature.

[2] Raviola E, Wiesel TN (1978). Effect of dark-rearing on experimental myopia in monkeys. Investigative Ophthalmology and Visual Science.

[3] Schaeffel F, Glasser A, Howland HC (1988). Accommodation, refractive error and eye growth in chickens. Vision Research.

[4] Smith EL, McGuire GW, Watson JT (1980). Axial lengths and refractive errors in kittens reared with an optically induced anisometropia. Investigative Ophthalmology and Visual Science.

[5] Wallman J, Turkel J, Trachtman J (1978). Extreme myopia produced by modest change in early visual experience. Science.

[6] Troilo D, Gottlieb MD, Wallman J (1987). Visual deprivation causes myopia in chicks with optic nerve section. Current Eye Research.

[7] Wildsoet CF, Pettigrew JD (1988). Experimental myopia and anomalous eye growth patterns unaffected by optic nerve section in chickens: Evidence for local control of eye growth. *Clinical Vision*. Sciences.

[8] Wallman J, Gottlieb MD, Rajaram V, Fugate-Wentzek L (1987). Local retinal regions control local eye growth and myopia. Science.

[9] Diether S, Schaeffel F (1997). Local changes in eye growth induced by imposed local refractive error despite active accommodation. Vision Research.

[10] Smith EL (2009). Hemiretinal form deprivation: evidence for local control of eye growth and refractive development in infant monkeys. Investigative Ophthalmology and Visual Science.

[11] Smith EL (2010). Effects of optical defocus on refractive development in monkeys: evidence for local, regionally selective mechanisms. Investigative Ophthalmology and Visual Science.

[12] Sherman SM, Norton TT, Casagrande VA (1977). Myopia in the lid-sutured tree shrew (Tupaia glis). Brain Res.

[13] Smith EL, Harwerth RS, Crawford MLJ, von Noorden GK (1987). Observations on the effects of form deprivation on the refractive status of the monkey. Investigative Ophthalmology and Visual Science.

[14] Troilo D, Judge S (1993). Ocular development and visual deprivation myopia in the common marmoset (Callithrix jacchus). Vision Research.

[15] Tigges M, Tigges J, Fernandes A, Eggers HM, Gammon JA (1990). Postnatal axial eye elongation in normal and visually deprived rhesus monkeys. Investigative Ophthalmology and Visual Science.

[16] Howlett MH, McFadden SA (2006). Form-deprivation myopia in the guinea pig (Cavia porcellus). Vision Research.

[17] Timmers AM, Fox DA, He LH, Hansen RM, Fulton AB (1999). Rod photoreceptor maturation does not vary with retinal eccentricity in mammalian retina. Current Eye Research.

[18] Sherman SM, Norton TT, Casagrande VA (1977). Myopia in the lid-sutured tree shrew *(Tupaia glis)*. Brain Research.

[19] Smith EL, Hung LF (2000). Form-deprivation myopia in monkeys is a graded phenomenon. Vision Research.

[20] Bartmann M, Schaeffel F (1994). A simple mechanism for emmetropization without cues from accommodation or colour. Vision Research.

[21] Bowrey HE, Metse AP, Leotta AJ, Zeng G, McFadden SA (2015). The relationship between image degradation and myopia in the mammalian eye. Clinical and Experimental Optometry.

[22] Feldkaemper M, Diether S, Kleine G, Schaeffel F (1999). Interactions of spatial and luminance information in the retina of chickens during myopia. Experimental Eye Research.

[23] Tran, N., Chiu, S., Tian, Y. & Wildsoet, C. F. The significance of retinal image contrast and spatial frequency composition for eye growth modulation in young chicks. *Vision Research* **48** (2008). 10.1016/j.visres.2008.03.022 PMC2712660 18533221

[24] Bassnett S, Beebee DC (1990). Localization of insulin-like growth factor-1 binding sties in the embryonic chicken eye. Investigative Ophthalmology and Visual Science.

[25] Troilo D, Quinn N, Baker K (2007). Accommodation and induced myopia in marmosets. Vision Research.

[26] Shaikh AW, Siegwart JT, Norton TT (1999). Effect of interrupted lens wear on compensation for minus lens in tree shrews. Optometry and Vision Science.

[27] Troilo D, Totonelly K, Harb EN (2009). Imposed anisometropia, accommodation, and regulation of refractive state. Optometry and Vision Sciences.

[28] Shapley R, Lennie P (1985). Spatial frequency analysis in the visual system. Annual Review of Neuroscience.

[29] Shapley R, Perry VH (1986). Cat and monkey retinal ganglion cells and their visual functional roles. Trends in Neurosciences.

[30] Siegwart JJ, Norton T (2010). Binocular lens treatment in tree shrews: Effect of age and comparison of plus lens wear with recovery from minus lens-induced myopia. Experimental Eye Research.

[31] Zhu X, Wallman J (2009). Temporal properties of compensation for positive and negative spectacle lenses in chicks. Investigative Ophthalmology and Visual Science.

[32] Winawer J, Zhu X, Choi J, Wallman J (2005). Ocular compensation for alternating myopic and hyperopic defocus. Vision Research.

[33] Leotta AJ, Bowrey HE, Zeng G, McFadden SA (2013). Temporal properties of the myopic response to defocus in the guinea pig. Ophthalmic and Physiological Optics.

[34] Norton TT, Siegwart JTJ, Amedo AO (2006). Effectiveness of hyperopic defocus, minimal defocus, or myopic defocus in competition with a myopiagenic stimulus in tree shrew eyes. Investigative Ophthalmology and Visual Science.

[35] Kee CS (2007). Temporal constraints on experimental emmetropization in infant monkeys. Investigative Ophthalmology and Visual Science.

[36] Benavente-Perez A, Nour A, Troilo D (2012). The effect of simultaneous negative and positive defocus on eye growth and development of refractive state in marmosets. Investigative Ophthalmology and Visual Science.

[37] Liu Y, Wildsoet C (2011). The effect of two-zone concentric bifocal spectacle lenses on refractive error development and eye growth in young chicks. Investigative Ophthalmology and Visual Science.

[38] Diether S, Wildsoet CF (2005). Stimulus requirements for the decoding of myopic and hyperopic defocus under single and competing defocus conditions in the chicken. Investigative Ophthalmology and Visual Science.

[39] Tse DY (2007). Simultaneous defocus integration during refractive development. Investigative Ophthalmology and Visual Science.

[40] Zhu X (2013). Temporal integration of visual signals in lens compensation (a review). Experimental Eye Research.

[41] Schmid KL, Wildsoet CF (1996). Effects on compensatory reponses to positive and negative lenses of intermittent lens wear and ciliary nerve section in chicks. Vision Research.

[42] Schmid KL, Wildsoet CF (1996). The sensitivity of the chick eye to refractive defocus. Ophthalmic and Physiological Optics.

[43] Flitcroft DI (2012). The complex interactions of retinal, optical and environmental factors in myopia aetiology. Progress in Retinal and Eye Research.

[44] Winawer J, Wallman J (2002). Temporal constraints on lens compensation in chicks. Vision Research.

[45] Nickla DL, Sharda V, Troilo D (2005). Temporal integration characteristics of the axial and choroidal responses to myopic defocus induced by prior form deprivation versus positive spectable wear in chickens. Optometry and Vision Science.

[46] Napper GA (1997). The effect of an interrupted daily period of normal visual stimulation on form deprivation myopia in chicks. Vision Research.

[47] Wildsoet CF (2019). IMI – Interventions for Controlling Myopia Onset and Progression ReportIMI – Interventions for Controlling Myopia. Investigative Ophthalmology & Visual Science.

[48] Benavente-Pérez A, Nour A, Troilo D (2014). Axial eye growth and refractive error development can be modified by exposing the peripheral retina to relative myopic or hyperopic defocus. Investigative Ophthalmology and Visual Science.

[49] Whatham AR, Judge SJ (2001). Compensatory changes in eye growth and refraction induced by daily wear of soft contact lenses in young marmosets. Vision Research.

[50] Benavente-Perez, A., Nour, A. & Troilo, D. Peripheral Refraction as a Predictor for Induced Changes in Vitreous Chamber Growth Rates in Marmosets. *Investigative Ophthalmology and Visual Science* **53**, E-abstract 4662 (2012).

[51] Benavente-Perez, A., Nour, A. & Troilo, D. Asymmetries in Peripheral Refraction in Marmosets Change with Emmetropization and Induced Eye Growth. *Investigative Ophthalmology and Visual Science* **55**, E-Abstract 2731 (2014).

[52] Gifford KL (2019). IMI – Clinical Management Guidelines Report. Investigative Ophthalmology & Visual Science.

[53] Siegwart JT, Norton TT (1998). The susceptible period for deprivation-induced myopia in tree shrew. Vision Research.

[54] Papastergiou GI (1998). Induction of axial eye elongation and myopic refractive error shift in one-year-old chickens. Vision Research.

[55] Smith EL, Bradley DV, Fernandes A, Boothe RG (1999). Form deprivation myopia in adolescent monkeys. Optometry and Vision Science.

[56] Troilo D, Nickla DL (2005). The response to visual form deprivation differs with age in marmosets. Investigative Ophthalmology and Visual Science.

[57] Norton TT, Amedo AO, Siegwart JT (2010). The effect of age on compensation for a negative lens and recovery from lens-induced myopia in tree shrews (Tupaia glis belangeri). Vision Res..

[58] Smith EL, Hung L-F, Huang J, Arumugam B (2013). Effects of local myopic defocus on refractive development in monkeys. Optom Vis Sci.

[59] Liu Y, Wildsoet C (2012). The effective add inherent in 2-zone negative lenses inhibits eye growth in myopic young chicks. Investigative Ophthalmology & Visual Science.

[60] Smith EL, Hung LF, Kee CS, Qiao Y (2002). Effects of brief periods of unrestricted vision on the development of form-deprivation myopia in monkeys. Investigative Ophthalmology and Visual Science.

[61] McBrien NA, Arumugam B, Metlapally S (2012). The effect of daily transient +4D positive lens wear on the inhibition of myopia in the tree shrew. Investigative Ophthalmology & Visual Science.

[62] Flitcroft DI, Knight-Nanan D, Bowell R, Lanigan B, O’Keefe M (1999). Intraocular lenses in children: changes in axial length, corneal curvature, and refraction. British Journal of Ophthalmology.

[63] Wallman J, Winawer J (2004). Homeostasis of eye growth and the question of myopia. Neuron.

[64] Kee CS (2007). NORTON. IOVS.

[65] Thibos LN, Bradley A, Liu T, López-Gil N (2013). Spherical aberration and the sign of defocus. Optometry and Vision Science.

