# Supplementary material for: Short Interruptions of Imposed Hyperopic Defocus Earlier in Treatment are More Effective at Preventing Myopia Development
Source: Sci Rep. 2019 Aug 7;9:11459. doi: 10.1038/s41598-019-48009-3 (PMC6685965; doi:10.1038/s41598-019-48009-3)
Supplement: Supplementary file 1 — Supplemental material Table 1 [file 41598_2019_48009_MOESM1_ESM.pdf]

Title of the manuscript: Short Interruptions of Imposed Hyperopic Defocus Earlier in Treatment are More Effective at Preventing Myopia Development

Authors:
*Alexandra Benavente-Perez MCOptom MS PhD
Ann Nour BS
David Troilo PhD
SUNY College of Optometry, New York, NY

	Treatment Group	Baseline OD	Baseline OS	4wks OD	4wks OS	8wks OD	8wks OS
Refractive Error (D, mean ± SE)	early interruption	-0.64 ± 0.68	-1.11 ± 0.76	-2.10 ± 0.62	-1.64 ± 0.51	-3.05 ± 0.57	-1.94 ± 0.46
	late interruption	-0.89 ± 0.83	-0.37 ± 0.39	-3.85 ± 0.69	-1.84 ± 0.45	-5.93 ± 0.61	-2.78 ± 0.42
	treated controls	2.18 ± 0.77	2.26 ± 0.61	-1.43 ± 1.10	0.43 ± 0.47	-3.58 ± 1.31	-0.88 ± 0.43
	untreated controls	0.03 ± 0.84	0.20 ± 0.71	-0.78 ± 0.50	-1.00 ± 0.60	-1.10 ± 0.36	-1.55 ± 0.40
Vitreous Chamber Depth (mm, mean ± SE)	early interruption	5.76 ± 0.04	5.74 ± 0.05	6.08 ± 0.05	6.05 ± 0.04	6.36 ± 0.05	6.27 ± 0.04
	late interruption	5.76 ± 0.04	5.74 ± 0.04	6.14 ± 0.04	6.02 ± 0.05	6.40 ± 0.04	6.12 ± 0.05
	treated controls	5.75 ± 0.13	5.75 ± 0.13	6.12 ± 0.11	6.03 ± 0.10	6.47 ± 0.11	6.31 ± 0.08
	untreated controls	5.77 ± 0.08	5.74 ± 0.08	6.10 ± 0.06	6.10 ± 0.07	6.33 ± 0.07	6.31 ± 0.08
Lens Thickness (mm, mean ± SE)	early interruption	2.15 ± 0.04	2.16 ± 0.05	2.07 ± 0.03	2.07 ± 0.03	2.00 ± 0.05	2.03 ± 0.04
	late interruption	2.12 ± 0.02	2.11 ± 0.02	2.04 ± 0.06	2.10 ± 0.02	1.99 ± 0.06	2.05 ± 0.02
	treated controls	2.05 ± 0.02	2.04 ± 0.02	2.03 ± 0.02	2.02 ± 0.02	1.97 ± 0.02	2.00 ± 0.02
	untreated controls	2.09 ± 0.03	2.09 ± 0.04	2.08 ± 0.02	2.08 ± 0.02	2.01 ± 0.02	2.01 ± 0.02
Anterior Chamber Depth (mm, mean ± SE)	early interruption	1.51 ± 0.03	1.50 ± 0.03	1.61 ± 0.03	1.59 ± 0.03	1.68 ± 0.03	1.66 ± 0.03
	late interruption	1.50 ± 0.01	1.51 ± 0.01	1.59 ± 0.01	1.58 ± 0.01	1.65 ± 0.01	1.64 ± 0.02
	treated controls	1.47 ± 0.03	1.48 ± 0.03	1.57 ± 0.03	1.58 ± 0.02	1.65 ± 0.02	1.65 ± 0.02
	untreated controls	1.48 ± 0.03	1.49 ± 0.03	1.55 ± 0.03	1.56 ± 0.03	1.66 ± 0.03	1.64 ± 0.03
Axial Length (mm, mean ± SE)	early interruption	9.42 ± 0.04	9.41 ± 0.03	9.76 ± 0.04	9.71 ± 0.03	10.04 ± 0.04	9.97 ± 0.03
	late interruption	9.37 ± 0.04	9.36 ± 0.06	9.77 ± 0.06	9.71 ± 0.04	10.05 ± 0.09	9.88 ± 0.05
	treated controls	9.27 ± 0.15	9.26 ± 0.15	9.72 ± 0.13	9.63 ± 0.10	10.09 ± 0.11	9.95 ± 0.08
	untreated controls	9.33 ± 0.11	9.32 ± 0.10	9.74 ± 0.09	9.74 ± 0.10	9.99 ± 0.10	9.97 ± 0.09
Retinal Thickness (mm, mean ± SE)	early interruption	0.22 ± 0.01	0.22 ± 0.01	0.25 ± 0.00	0.23 ± 0.01	0.23 ± 0.01	0.24 ± 0.00
	late interruption	0.23 ± 0.00	0.23 ± 0.00	0.23 ± 0.00	0.22 ± 0.01	0.23 ± 0.01	0.24 ± 0.00
	treated controls	0.24 ± 0.00	0.24 ± 0.00	0.22 ± 0.01	0.24 ± 0.00	0.23 ± 0.01	0.23 ± 0.01
	untreated controls	0.23 ± 0.00	0.24 ± 0.00	0.22 ± 0.01	0.22 ± 0.01	0.23 ± 0.01	0.24 ± 0.01
Choroidal Thickness (mm, mean ± SE)	early interruption	0.12 ± 0.00	0.12 ± 0.01	0.12 ± 0.00	0.11 ± 0.01	0.13 ± 0.01	0.14 ± 0.00
	late interruption	0.12 ± 0.00	0.13 ± 0.01	0.12 ± 0.01	0.13 ± 0.01	0.13 ± 0.01	0.13 ± 0.01
	treated controls	0.12 ± 0.01	0.12 ± 0.02	0.11 ± 0.01	0.11 ± 0.01	0.13 ± 0.01	0.12 ± 0.00
	untreated controls	0.12 ± 0.01	0.11 ± 0.01	0.12 ± 0.01	0.12 ± 0.01	0.12 ± 0.00	0.13 ± 0.00
Corneal Curvature (mm, mean ± SE)	early interruption	3.45 ± 0.04	3.48 ± 0.02	3.47 ± 0.05	3.48 ± 0.05	3.51 ± 0.03	3.45 ± 0.04
	late interruption	3.48 ± 0.02	3.50 ± 0.02	3.49 ± 0.02	3.48 ± 0.03	3.54 ± 0.02	3.54 ± 0.02
	treated controls	3.42 ± 0.04	3.41 ± 0.04	3.46 ± 0.02	3.44 ± 0.03	3.48 ± 0.03	3.44 ± 0.02
	untreated controls	3.54 ± 0.06	3.54 ± 0.06	3.59 ± 0.06	3.57 ± 0.06	3.61 ± 0.06	3.59 ± 0.06

Supplemental Table S1. Ocular biometry and refractive state for the experimental (OD) and control eyes (OS) after 4 and 8 weeks of treatment. The data are shown as mean±SE.
