# Supplementary material for: Short Interruptions of Imposed Hyperopic Defocus Earlier in Treatment are More Effective at Preventing Myopia Development
Source: Sci Rep. 2019 Aug 7;9:11459. doi: 10.1038/s41598-019-48009-3 (PMC6685965; doi:10.1038/s41598-019-48009-3)
Supplement: Supplementary file 2 — Supplemental material Table 2 [file 41598_2019_48009_MOESM2_ESM.pdf]

Title of the manuscript: Short Interruptions of Imposed Hyperopic Defocus Earlier in Treatment are More Effective at Preventing Myopia Development

Authors:
*Alexandra Benavente-Perez MCOptom MS PhD
Ann Nour BS
David Troilo PhD
SUNY College of Optometry, New York, NY

	Treatment Group	Changes from 0-4wks OD	Changes from 0-4wks OS	Changes from 4-8wks OD	Changes from 4-8wks OS
Refractive Error (D, mean ± SE)	early interruption	-1.46 ± 1.00	-0.53 ± 1.04	-2.41 ± 1.04	-0.82 ± 0.89
	late interruption	-2.96 ± 0.34	-1.47 ± 0.50	-5.04 ± 0.58	-2.41 ± 0.61
	treated controls	-3.61 ± 0.67	-1.83 ± 0.43	-5.76 ± 0.94	-3.14 ± 0.60
	untreated controls	-0.81 ± 0.55	-1.20 ± 0.33	-1.14 ± 0.60	-1.75 ± 0.51
Vitreous Chamber Depth (mm, mean ± SE)	early interruption	0.32 ± 0.03	0.30 ± 0.02	0.60 ± 0.03	0.53 ± 0.03
	late interruption	0.39 ± 0.02	0.28 ± 0.02	0.65 ± 0.04	0.45 ± 0.03
	treated controls	0.37 ± 0.03	0.28 ± 0.04	0.72 ± 0.06	0.56 ± 0.06
	untreated controls	0.34 ± 0.05	0.36 ± 0.04	0.56 ± 0.06	0.57 ± 0.04
Lens Thickness (mm, mean ± SE)	early interruption	-0.08 ± 0.02	-0.09 ± 0.04	-0.15 ± 0.03	-0.14 ± 0.04
	late interruption	-0.09 ± 0.04	-0.02 ± 0.01	-0.13 ± 0.05	-0.07 ± 0.02
	treated controls	-0.03 ± 0.01	-0.02 ± 0.01	-0.08 ± 0.01	-0.04 ± 0.02
	untreated controls	0.00 ± 0.02	-0.01 ± 0.02	-0.07 ± 0.03	-0.08 ± 0.02
Anterior Chamber Depth (mm, mean ± SE)	early interruption	0.10 ± 0.01	0.09 ± 0.03	0.17 ± 0.02	0.17 ± 0.03
	late interruption	0.09 ± 0.01	0.07 ± 0.01	0.16 ± 0.01	0.13 ± 0.01
	treated controls	0.10 ± 0.01	0.10 ± 0.01	0.18 ± 0.02	0.17 ± 0.02
	untreated controls	0.08 ± 0.02	0.07 ± 0.01	0.18 ± 0.02	0.16 ± 0.02
Axial Length (mm, mean ± SE)	early interruption	0.34 ± 0.03	0.30 ± 0.02	0.62 ± 0.03	0.56 ± 0.03
	late interruption	0.39 ± 0.05	0.34 ± 0.02	0.68 ± 0.08	0.52 ± 0.03
	treated controls	0.45 ± 0.04	0.36 ± 0.05	0.81 ± 0.07	0.68 ± 0.08
	untreated controls	0.41 ± 0.05	0.42 ± 0.06	0.66 ± 0.06	0.65 ± 0.05
Retinal Thickness (mm, mean ± SE)	early interruption	0.03 ± 0.01	0.01 ± 0.01	0.01 ± 0.01	0.02 ± 0.01
	late interruption	0.00 ± 0.01	-0.01 ± 0.01	0.00 ± 0.01	0.00 ± 0.01
	treated controls	-0.01 ± 0.01	0.00 ± 0.00	-0.01 ± 0.00	0.00 ± 0.01
	untreated controls	-0.01 ± 0.01	-0.02 ± 0.01	0.00 ± 0.01	0.00 ± 0.00
Choroidal Thickness (mm, mean ± SE)	early interruption	0.00 ± 0.00	0.00 ± 0.01	0.01 ± 0.01	0.02 ± 0.01
	late interruption	0.00 ± 0.01	0.00 ± 0.00	0.01 ± 0.01	0.01 ± 0.01
	treated controls	-0.01 ± 0.02	-0.01 ± 0.02	0.01 ± 0.01	0.00 ± 0.02
	untreated controls	0.00 ± 0.01	0.01 ± 0.01	0.00 ± 0.01	0.01 ± 0.01
Corneal Curvature (mm, mean ± SE)	early interruption	0.02 ± 0.06	0.00 ± 0.04	0.06 ± 0.04	-0.03 ± 0.04
	late interruption	0.00 ± 0.03	-0.02 ± 0.04	0.05 ± 0.03	0.04 ± 0.01
	treated controls	0.04 ± 0.03	0.03 ± 0.02	0.06 ± 0.04	0.03 ± 0.02
	untreated controls	0.05 ± 0.02	0.03 ± 0.03	0.06 ± 0.02	0.04 ± 0.02

Supplemental Table S2. Changes in ocular biometry and refractive state for the experimental (OD) and control eyes (OS) after 4 and 8 weeks of treatment. The data are shown as mean±SE
